# ASO Author Reflections: The Need for Improvement of the 8th American Joint Committee on Cancer TNM Staging System for Resected Pancreatic Ductal Adenocarcinoma

**DOI:** 10.1245/s10434-022-11738-3

**Published:** 2022-04-19

**Authors:** Thijs J. Schouten, Lois A. Daamen, Hjalmar C. van Santvoort, I. Quintus Molenaar

**Affiliations:** Department of Surgery, Regional Academic Cancer Center Utrecht, UMC Utrecht Cancer Center and St. Antonius Hospital Nieuwegein, Utrecht, Netherlands

## Past

Recently, the 8th edition of the American Joint Committee on Cancer (AJCC) tumor–node–metastasis (TNM) staging system for resected pancreatic ductal adenocarcinoma (PDAC) was introduced into clinical practice.^1^ Although it showed improved discriminative power as compared with the 7th edition, it has been validated in high-volume pancreatic centers only. Consequently, its general applicability could be questioned. In addition, four recent studies have proposed modifications of the 8th edition. These modifications demonstrated a further increase in prognostic accuracy but lack external validation.^2–5^

## Present

In this study, the prognostic value of the 8th AJCC TNM classification and proposed modifications was evaluated in a cohort of 750 consecutive patients who underwent PDAC resection in the Netherlands between 2014 and 2016.^6^ The 8th edition distributed patients more equally over all disease stages as compared with the 7th edition and displayed increased prognostic accuracy (C-index 0.59 versus 0.56, respectively). In our cohort, the proposed modifications did not further improve its prognostic value. We developed a new classification, migrating T3N1 patients to stage III, which resulted in a more even distribution of patients. The new modification also showed a C-index of 0.59 but demonstrated significant survival differences between all TNM stages (*P *< 0.05). Consequently, it allowed for better prognostication in patients with all disease stages as compared with former classifications.

## Future

Accurate prediction of survival for individual patients is crucial to correctly inform patients on their prognosis and can be helpful in the shared decision-making process regarding the direction of treatment decisions. Nevertheless, prognosis in PDAC patients remains hard to predict due to heterogenic tumor biology. Future studies should therefore seek more powerful predictors that can be incorporated into the TNM staging system. However, this must not reduce the simplicity of the TNM staging system, which allows doctors to communicate globally using a standardized language that reflects tumor burden.
